# Effect of Pretreatment Processes on Biogenic Amines Content and Some Bioactive Compounds in *Hericium erinaceus* Extract

**DOI:** 10.3390/foods10050996

**Published:** 2021-05-02

**Authors:** Netnapa Makhamrueang, Sasithorn Sirilun, Jakkapan Sirithunyalug, Wantida Chaiyana, Wiwat Wangcharoen, Sartjin Peerajan, Chaiyavat Chaiyasut

**Affiliations:** 1 Innovation Center for Holistic Health, Nutraceuticals, and Cosmeceuticals, Faculty of Pharmacy, Chiang Mai University, Chiang Mai 50200, Thailand; netnapa_ma@cmu.ac.th (N.M.); wantida.chaiyana@cmu.ac.th (W.C.); 2 Department of Pharmaceutical Sciences, Faculty of Pharmacy, Chiang Mai University, Chiang Mai 50200, Thailand; jakkapan.s@cmu.ac.th; 3 Department of Food Technology, Faculty of Engineering and Agro-Industry, Maejo University, Chiang Mai 50290, Thailand; wiwat@mju.ac.th; 4 Health Innovation Institute, Chiang Mai 50200, Thailand; s.peerajan@gmail.com

**Keywords:** biogenic amine, β-glucan, *Hericium erinaceus*, pretreatment, antioxidant activity

## Abstract

*Hericium erinaceus* is reported as a source of several nutritional contents and bioactive compounds, especially β-glucan. However, various uncontrolled processes lead to the formation of byproducts that can affect human health, including biogenic amines. These amines are concerning, because their presence is an important indicator of the process of hygiene and food spoilage or quality. A better understanding of various pretreatment processes can control the content of biogenic amines. In this work, we studied the effect of pretreatment processes, i.e., sample size (whole, ripping, and chopping); heating process (non-heating, blanching, and boiling); and drying method (nondrying, hot air drying, and freeze-drying) on biogenic amine contents in *H. erinaceus* extract. A method of the post-column high-performance liquid chromatography (HPLC) technique was used for the analysis of putrescine (PUT) and spermidine (SPD) in *H. erinaceus* extract following the acceptable guidelines. In this study, treatment 20 (chopping/non-heating/hot air drying) was suggested as a good choice for the pretreatment process, because low levels of PUT and SPD were shown in the extract while high levels of the bioactive compounds β-glucan and antioxidant activity were presented. This treatment process can be applied to the industry because of its easy operation and cost-saving.

## 1. Introduction

*Hericium erinaceus*, also known as Monkey head mushroom, Lion’s Mane, Houtou (Chinese), and Yamabushitake (Japanese), is an edible mushroom and medicinal mushroom. Several studies have reported that they are rich with bioactive compounds and polysaccharides such as antioxidative agents, polyphenol, and β-glucan [1,2]. Especially, β-glucan is found as the main representative of polysaccharides in *H. erinaceus* [3,4]. They are responsible for the immunomodulating, neuroprotective activity, hypolipidemic, antioxidant, and anticancer properties [1,2,3,4,5]. *H. erinaceus* is generally used in several processed food products, such as dried, powder, capsule, juice, and fermented mushroom beverages. However, various uncontrolled processes lead to the formation of undesirable compounds that can affect human health, i.e., ethanol, methanol, and some toxins, including biogenic amine. Some uncontrolled processes might occur from the contamination of microorganisms from equipment or workers. Yeast can convert sugar (contain or add in foods) to alcohol during the fermentation process [6]. A slice of ham was characterized with a higher tyramine (TYR) content when kept at a high temperature [7]. A study of the TYR content in fermented sausages found that an increased salt concentration (from 0% to 5% of NaCl) reduced the TYR concentration [8]. Santos et al. [9] studied the size reduction of meat in hamburgers. They reported that biogenic amine, TRY, increased with the contamination because of grinding meat. To our knowledge, the most factors to reduce biogenic amines in foods are the suitable hygienic conditions of raw material and manufacturing or storage processes. The use of selected decarboxylase-negative bacteria in fermentation products also leads to low biogenic amine accumulation. Biogenic amines are usually generated by the microbial through decarboxylation of the corresponding amino acids. Although, biogenic amines in foods are associated with three main factors, including (i) the presence of amino acid as a precursor, (ii) the availability of various microorganism that can decarboxylase amino acid, and (iii) the environment conditions of a microorganism that permits the growth and production of the enzyme [10,11,12,13]. These compounds are found in many foods, including beverages, which can cause adverse effects to the consumer health. The safety criteria of histamine in fishery products are regulated as 100–400 mg/kg by the European Community [14] and 50 mg/kg by the US Food and Drug Administration [15]. At the same time, the information was insufficient for other biogenic amines and other foods different from fish. High intakes of toxic biogenic amines may lead to rashes, flushing, nausea, vomiting, diarrhea, headaches, migraines, cardiac palpitations, and blood pressure changes [16,17]. In the food industry, the contents of the biogenic amines were interesting as an indicator of the hygiene process and food spoilage or quality for monitoring of the processing and storage period. Currently, there are many techniques for an appropriate biogenic amines analysis, including suitable sample extraction. High-performance liquid chromatography (HPLC) is the favorite analysis method because of the low volatility. This method lacks the chromophores of most biogenic amine derivatization procedures [18]. Lavizzari et al. [19] studied the HPLC analysis to determine the selected biogenic amines in vegetal food products, such as spinach, hazelnuts, bananas, potatoes, and chocolate milk. The post-column derivatization with ortho-phthalaldehyde (OPA) as a reagent allowed the identification of these amines. The derivatization was not affected by the sample matrix during the reaction with the reagent due to the sampling components being separated in the column prior to the reaction.

In this study, putrescine (PUT) and spermidine (SPD) were the considered biogenic amines due to a high content in the mushrooms. [10,20,21]. Sanchez-Pere et al. [22] suggested that there are 0–156 mg of putrescine (PUT) per kg of fresh mushroom and 9–155 mg of spermidine (SPD) per kg of fresh mushroom. In another study, the amounts of PUT and SPD were 51 and 344 nmol per g, respectively [23]. Dadáková et al. [21] studied the biogenic amines in wild-growing edible mushrooms. *H. erinaceus* was one of the samples. The results showed that the highest content of amine was PUT 150 mg per kg of fresh matter. This mushroom also contained a high level of SPD as 100 mg per kg of fresh matter. However, there is limited literature on the report of the biogenic amine content for *H. erinaceus*. They provide information on the amine accumulation of the *H. erinaceus* mushroom: PUT was 140 nmol per kg (12.3 mg/kg wet weight), and SPD was 493 nmol per kg (71.5 mg/kg wet weight) [21,24]. Accordingly, PUT and SPD were the amines that were mainly considered in the study of *H. erinaceus* and their products.

A better understanding of the various pretreatment processes can control the formation of biogenic amines. Blanching is a popular pretreatment process for vegetables. The objective of blanching produces specific favorable effects on processed materials, such as the inactivation of enzymes and removal of undesirable flavors and aromas [25]. However, there are some limitations, e.g., vitamin loss, nutrient leaching, and texture changes. Other modes of processing may also be performed on a pretreated material, such as boiling. This process may enhance the release of the interested compound from the cell of the material. Boiling can inhibit some enzymatic substances. Paulsen and Bauer [26] reported that thermal processing—namely, boiling and grilling—can decrease SPM (spermine) and SPD up to 26% in pork loins, combined with 20% brine curing and storage at 4 °C. Hong et al. [27] studied the freezing treatment on big head fish, affecting the biogenic amine (SPM and SPD) reduction. The three different freezing conditions, i.e., (i) at −40 °C, (ii) at −18 °C, and (iii) at −40 °C for 12 h and then −18 °C, were tested to store the sample for three months. As a result, SPM and SPD were significantly reduced compared with the fresh sample. The time and temperature of storage are the ones that influence bacterial-producing biogenic amine growth during food processing and storage. The report kept the sample at the temperature below 5 °C; these methods delay the formation of biogenic amine in food, primarily through the inhibition of bacteria or the decarboxylase enzyme activity responsible for biogenic amine formation [14]. It should be noted that these amines are very difficult to destroy by processing [28,29].

From the point of safety, it is important to understand how the pretreatment process influences the alterations of biogenic amine in *H. erinaceus.* The purpose of this study is that pretreatment processes can present a low accumulation of PUT and SPD in the *H. erinaceus* extract while maintaining bioactive compounds such as β-glucan and antioxidant activity. Three main factors were studied, including the sample size (whole, ripping, and chopping); heating process (non-heating, blanching, and boiling); and drying method (nondrying, hot air drying, and freeze-drying). The analysis method studied the biogenic amine in the *H. erinaceus* extract using the post-column high-performance liquid chromatography (HPLC) technique.

## 2. Materials and Methods

### 2.1. Chemicals

Perchloric acid was obtained from Loba Chemie (Mumbai, India). The standard of biogenic amines, putrescine dihydrochloride and spermidine trihydrochloride, were obtained from Sigma-Aldrich (St. Louis, MO, USA). Water, methanol, and acetonitrile of HPLC grade were obtained from RCl Labscan (Bangkok, Thailand). The other reagents for synthesis were: sodium acetate anhydrous, purchased from QReC (Selangor, Malaysia); sodium 1-octanesulfonate monohydrate, purchased from Sigma-Aldrich (St. Louis, MO, USA); boric acid and glacial acetic acid, obtained from RCl Labscan (Bangkok, Thailand); potassium hydroxide, purchased from Kemaus (Cherrybrook, New South Wales, Australia); Brij 35, purchased from Merck (Billerica, MA, USA); and 2-mercaptoethanol, purchased from Merck (Darmstadt, Germany). OPA was obtained from Himedia (Mumbai, India).

### 2.2. Material

*H. erinaceus* was obtained from a contact farm in Doi Saket District, Chiang Mai, Thailand. The mushroom was harvested in a 10–12-day cycle. The raw materials were kept at −20 °C until the experimental period. The mushrooms were washed under running tap water and drained on a screen to eliminate excess water before the experiment.

### 2.3. Pretreatment Processes of H. erinaceus by the Design of Experiment

The experimental design was set as a full factorial in a completely randomized design (CRD). Factorial experimentations with triplicate replication (3^3^), leading a total of 81 runs, were carried out for 3 independent variables, including the sample size (X_1_), heating process (X_2_), and drying method (X_3_). The code level of the variable at −1, 0, and 1 is shown in Table 1. The experimental design results were determined by an analysis of variance (ANOVA) based on the *p*-value at the 95% confidence level.

*H. erinaceus* was operated by 3 factors, including the sample size (whole, ripping, and chopping); heating process (non-heating, blanching, and boiling); and drying method (nondrying, hot air drying, and freeze drying). In the case of a sample size of fresh *H. erinaceus*, the mushroom was ripped up by hand to obtain a sample about the dimensions of 0.1 × 5 cm (width × length) or chopped to small pieces by a blender (Blend-Xtract 3-in-1 Blende, BL237WG, Irving, TX, USA) for 2 min. Samples were blanched at 100 °C for 1 min or boiled at 100 °C for 5 min and measured at the start of boiling [30]. After blanching and boiling, the sample was quickly cooled in cold water (4 °C) [31]. For the drying method, the mushroom samples were subjected to either hot air drying or freeze-drying. In the case of hot air drying, a sample was dried in a laboratory-scale hot air dryer (Drawell, DGT-G135, Shanghai, China). Five hundred grams of the sample was spread on a tray with the dimensions of 25 × 40 cm, and then, the samples were dried at 60 °C until a final moisture content of less than 10% dry weight was reached. In the case of freeze-drying, 200 g of a sample was spread on a tray with the dimensions of 25 × 40 cm, which was located in the freezing dryer (Drawell, DW-F30N, Shanghai, China). The dried sample was packed in an aluminum packet and kept in a desiccator at room temperature until further use.

### 2.4. Biogenic Amines Extraction from H. erinaceus

The extraction method was referred from Lavizzari et al. [19]. Twenty-seven pretreated mushrooms were ground using a blender (Blend-Xtract 3-in-1 Blende, BL237WG, Irving, TX, USA) for 1 min. One gram of fresh sample or five grams of dried sample was accurately weighed in a 50-mL centrifuge tube. The sample was then extracted with 10 mL of 0.6-M perchloric acid in a magnetic stirring plate for 20 min. The solution was centrifuged at 12,000× *g* at 4 °C for 30 min. The supernatant of the extract was collected in the tube, and the pellet was mixed twice with 7 mL of 0.6-M perchloric acid. The final combined supernatant was adjusted to 25 mL with 0.6-M perchloric acid. The extract was filtered through a 0.45-µm nylon filter (Nylon membrane, Millipore, Bedford, MA, USA) and kept in an amber glass bottle for analysis.

### 2.5. Method Validation of Biogenic Amine Analysis in H. erinaceus Extract

This validated method was a specifically proven PUT and SPD analysis in *H. erinaceus* related to the current HPLC equipment. The biogenic amines analysis was validated with regards to the parameters of accuracy, precision, linearity, limit of detection (LOD), and limit of quantitation (LOQ). The validated parameters were referred to according to the International Council for Harmonization (ICH) [32] and Guideline and Association of Official Analytical Chemists (AOAC) [33].

Linearity was assessed by least-squares fitting seven-point calibration curves at varying concentration levels of biogenic amine mixture standards: 0.10, 0.50, 1.00, 2.50, 5.00, 10.00, and 20.00 mg/L. The concentration points were assayed in triplicate. Linear curves were plotted between the peak area and the concentrations of PUT and SPD; the results were reported as correlation coefficients. The acceptable requirement was in the range of 0.995–1.000.

The accuracy of the method was evaluated by spiking each biogenic amine at three concentrations (80%, 100%, and 120% of the extract sample concentration), performing three replications for each concentration level. The results were reported as a percentage of the recovery that could be calculated by comparing the amount of biogenic amine detected in a sample with the amount of standard material added to the sample, as shown in Equation (1):

% recovery = (C_f_ − C_u_) × 100/C_a_, (1)

where C_f_ is the concentration of the fortified sample, C_u_ is the concentration of the unfortified sample, and C_a_ is the concentration of the standard material added to the sample.

The precision value was given as the percentage of relative standard deviation (RSD) of the concentration in which intraday precision and inter-day precision were studied in this work. Intraday precision was assessed by spiking the standard mixtures of two biogenic amines at three concentrations (80%, 100%, and 120% of extract sample concentration), with seven replications in the same day. Meanwhile, the inter-day precision was done daily by spiking the standard mixtures of two biogenic amines at three concentrations (80%, 100%, and 120% of extract sample concentration) with seven replications for three days.

The determination of the limit of detection (LOD) and limit of quantification (LOQ) was carried out by injecting the standard mixtures of two biogenic amines at the concentrations of 80%, 100%, and 120% of the *H. erinaceus* extract sample concentration with seven replications. The LOD and LOQ were calculated on the basis of the standard deviation of the response, and the slope was obtained from the calibration curve, as described in ICH Q2(R1) [32]. The results can be expressed as Equations (2) and (3), respectively.

LOD = 3.3 σ/S,(2)

LOQ = 10 σ/S,(3)

where σ is the standard deviation of the response, and S is the slope of the calibration curve.

### 2.6. Biogenic Amines Analysis Using Post-Column Derivatization High-Performance Liquid Chromatography (HPLC)

The HPLC analysis was optimized and modified according to the post-column derivatization HPLC technique of Lavizzari et al. [19]. A system controller (model SCL-10A VP, Shimadzu, Tokyo, Japan), two auto-pumps (model LC-10AS, Shimadzu, Tokyo, Japan), a spectrofluorometric detector (model SCL-10A VP, Shimadzu, Tokyo, Japan), and a post-column pump (Waters, Milford, MA, USA), which connected the column outlet and the detector with T mixing, were used. The filtrate was injected into a C18 reversed-phase column (4 µm, 4.6 × 150 mm, Poroshell 120 EC-C18, Agilent Technologies, Santa Clara, CA, USA). The column was placed into a column oven (model CTO-10AS, Shimadzu, Tokyo, Japan) to keep a constant temperature at 40 °C.

The mobile phases A and B were a mixture of sodium acetate and sodium octanesulfonate adjusted to the pH with acetic acid. The mobile phase A was 50% (*v/v*) of 0.1-M sodium acetate and 50% (*v/v*) of 10-mM sodium 1-octanesulfonate monohydrate, in which the mixture of mobile phase A was adjusted to pH 5.23 with glacial acetic acid. The composition of mobile phase B was composed of 33% (*v/v*) of 0.2-M sodium acetate, 33% (*v/v*) of 10-mM sodium 1-octanesulfonate monohydrate, and 34% (*v/v*) of acetonitrile. The combined solvent of mobile phase B was adjusted to pH 4.50 with glacial acetic acid. A pH meter was used to control the pH of the mobile phases. The mixed mobile phase passed through a 0.45-μm nylon filter and then was degassed for 40 min in an ultrasonic bath (Trassonic Digital S, Elma, Singen, Germany) before the run. The flow rate of the mobile phases was 1.2 mL/min in the gradient program, as shown in Table 2.

In part of the post-column derivatization reagent, the solution was freshly prepared. Firstly, 31 g of boric acid and 26 g of potassium hydroxide were accurately weighed and diluted in 1000 mL of HPLC water. The reducing agent, 5 mL of 30% Brij 35 and 5 mL of 2-mercaptoethanol, was added to the solution. A small amount of OPA (0.2 g) was dissolved with 5 mL of methanol and then added to the solution. The derivatization reagent was filtered through a 0.45-μm nylon filter and then degassed in an ultrasonic bath. The reagent was kept in an amber bottle and the dark before further analysis. The flow rate of the mobile phases was 0.6 mL/min. The biogenic amines were detected by a fluorometric detector after post-column derivatization at 340 nm for excitation and 445 nm for emission.

The biogenic amine contents were reported as µg/g dry weight due to the changes in the dry matter contents during processing. The dry weight contents were determined in a homogenized sample using hot air oven drying at 105 °C until the samples did not have a difference in weights.

### 2.7. β-glucan Analysis

β-glucan was analyzed by a mushroom and yeast β-glucan assay kit K-YBGL 08/18 (Megazyme, Wicklow, Ireland). A UV-Vis spectrophotometer measured the reaction at a wavelength of 510 nm. The β-glucan concentration in the samples was calculated by the total glucan minus α-glucan.

### 2.8. ABTS Radical Cation Scavenging Activity

The ABTS assay was described by Pumtes, P. et al. (2016) [34]. ABTS^•+^ radical cation was prepared from 2 mL of 7-mM aqueous stock solution of ABTS and 1 mL of 2.45-mM potassium persulfate. The solution was kept in the dark at room temperature for 16 h. The solution was then diluted with ethanol to an absorbance of 0.70 (±0.02) at 734 nm. Some (160 μL) ABTS^•+^ solution and 40 μL of extract were mixed. The absorbance was measured at 734 nm using the spectrophotometer. Pure ethanol was again used to calibrate the spectrophotometer. The ABTS radical cation scavenging activity or antioxidant activity were also quantified using Trolox as the standard.

### 2.9. Statistics Analysis

The data are presented as mean values with standard deviations. Differences between mean values were established using Tukey’s test; values were considered at a confidence level of 95%. All statistical analyses were performed using SPSS (version 17, SPSS Inc., Chicago, IL, USA). All experiments were performed in triplicate unless specified otherwise.

## 3. Results

### 3.1. Method Validation of Biogenic Amine in H. erinaceus Extract

The results of the validation parameters are shown in Table 3. The calibration curve of the two biogenic amines exhibited excellent linearity over the tested range. The coefficient of determination (R^2^) of PUT and SPD were 0.9995 and 0.9981, respectively, which were in the acceptable requirements (0.995–1.000) [33]. The percent of recovery was within the range from 100.47% to 107.76%, which is considered acceptable for the criteria (80–115%) according to the AOAC guidelines [33]. These results clearly showed that the accuracy for determining the biogenic amines in the *H. erinaceus* extract sample was in the acceptable recovery requirements.

The RSD of intraday precision for the peak area ranged from 4.27% to 5.86%. Meanwhile, the RSD of inter-day precision was in the range of 4.00–5.72%. As a result, the precision is considered acceptable for the criteria (intraday precision < 6% and inter-day precision < 11%) according to the AOAC guidelines [33]. These results clearly showed that the precision for the determination of the biogenic amines in the *H. erinaceus* extract sample was in the acceptable requirements.

As shown in Table 3, the resulting LODs for PUT and SPD were 0.01 and 0.55 mg/mL, respectively, and the LOQs for PUT and SPD were 0.04 and 1.67 mg/mL, respectively.

### 3.2. Biogenic Amines Content in H. erinaceus Extract with Different Pretreatment Processes

The amounts of the biogenic amines in the *H. erinaceus* extracts with the different pretreatment processes (27 treatments) are shown in Table 4. The PUT and SPD contents were in the range of 0.81–83.16 µg/g dry weight and 3.53–148.74 µg/g dry weight, respectively. The minimum PUT content was treatment 23 with chopping, blanching, and hot air drying, while the minimum SPD content was observed in treatment 15 with ripping, blanching, and freeze-drying. The maximum PUT and SPD contents were observed in treatment 4 (whole, blanching, and nondrying) and treatment 7 (whole, boiling, and nondrying), respectively.

### 3.3. β-glucan Content in H. erinaceus Extract with Different Pretreatment Processes

**Figure 1:**
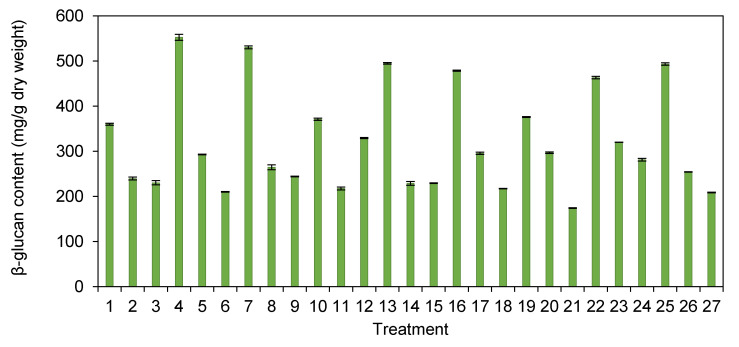
The contents of β-glucan in the *H. erinaceus* extracts from different pretreatment processes (Error bars represent a standard deviation of three replicates).

Figure 1 showed the contents of β-glucan in the *H. erinaceus* extracts from various treatments. This study found that the β-glucan contents of the extracts from *H. erinaceus* treated by different pretreatment processes were in the range of 173.99–552.52 mg/g dry weight. Treatment 21 (chopping/non-heating/freeze-drying) had the lowest level of β-glucan. The maximum β-glucan content was indicated by treatment 4 with the whole sample size, blanching, and nondrying.

### 3.4. Antioxidant Activity in H. erinaceus Extract with Different Pretreatment Processes

**Figure 2:**
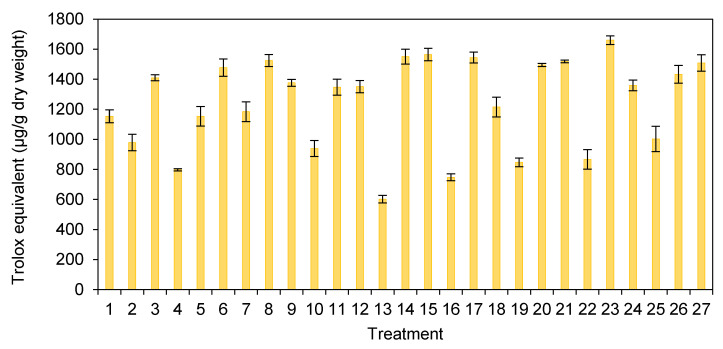
The Trolox equivalent in the *H. erinaceus* extracts from different pretreatment processes (Error bars represent a standard deviation of three replicates).

The ABTS assays assessed the antioxidant activity of the extracts prepared from *H. erinaceus* at different pretreatment processes. They were in the range of 601.94–1659.84 µg Trolox equivalent/g dry weight (Figure 2). Treatment 13 (ripping/blanching/nondrying) showed the lowest level of antioxidant activity, and treatment 23 (chopping/blanching/hot air drying) presented the highest level of antioxidant activity.

## 4. Discussion

Many studies have been performed to study the comparison of pre-column and post-column HPLC derivatization methods. Simat and Dalgaard [35] compared the pre-column and post-column derivatization for a biogenic amines analysis in seafood (lean canned tuna and fatty frozen herring). The time of elution, eluent consumption, backpressure, separation, sensitivity, recovery, and repeatability were determined. The results showed that separation, sensitivity, recovery, and repeatability for post-column derivatization were better than pre-column derivatization because of the reduced time of analysis and eluent consumption. These results confirmed that this method is an alternative to the biogenic amine analysis for other foods. Therefore, the biogenic amines (PUT and SPD) were analyzed by the modified method of the post-column derivatization HPLC following Lavizzari et al. [19]. Due to the different equipment and environments from the reference method, the gradient elution program was optimized to get a stable and good separation of the peak resolutions in this study. The reliability of the post-column derivatization HPLC analysis was considered using the validation parameters, including linearity, accuracy, precision, LOD, and LOQ. The modified method of the post-column derivatization HPLC was a suitable analysis of the biogenic amines PUT and SPD in the *H. erinaceus* extract sample following the acceptable guidelines.

In this study, two biogenic amines (PUT and SPD) were analyzed from the *H. erinaceus* extract, because they were the most abundant compounds and existed at a high level in *H. erinaceus* mushrooms and other mushrooms [21,22,23,24,36]. In the case of this study, the PUT and SPD contents of the untreated *H. erinaceus* mushrooms were 10.32 and 102.52 µg/g dry weight, respectively, as shown in treatment 1 (whole/non-heating/nondrying) during our pretreatment processes. These are in the range of the previous literature. Schindler, B.K. et al. (2015) suggested that there was 0–1010 mg of putrescine (PUT) per kg of dry weight [37]. Sánchez-Pérez, S et al. (2018) reported 0–156 mg of putrescine (PUT) per kg of fresh mushroom and 9–155 mg of spermidine (SPD) per kg of fresh mushroom [22]. This is not surprising; the *H. erinaceus* samples came from different sources and pretreatments; the contents of the biogenic amines can have a broad range. Accordingly, controlling the raw materials is an important factor that concerns the contents of the biogenic amines, which indicates the quality of the food products. Interesting, for future work, is how to control biogenic amine contents during plant growing and harvesting.

Considering the suitable pretreatment processes for a low level of biogenic amine contents in the *H. erinaceus* extract, a 3^3^ factorial in CRD was used as the tool for determination. The design inspected the main factors and the interactions between the factors, such as the sample size, heating process, and drying method, for the PUT and SPD contents. The statistical analysis was applied to explain the relationships between the main factors and their interactions. The results showed that the three variable factors significantly affected both of the biogenic amines in the *H. erinaceus* extract (*p* < 0.05).

**Figure 3:**
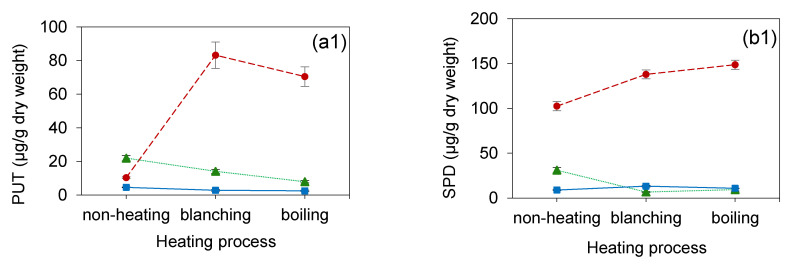

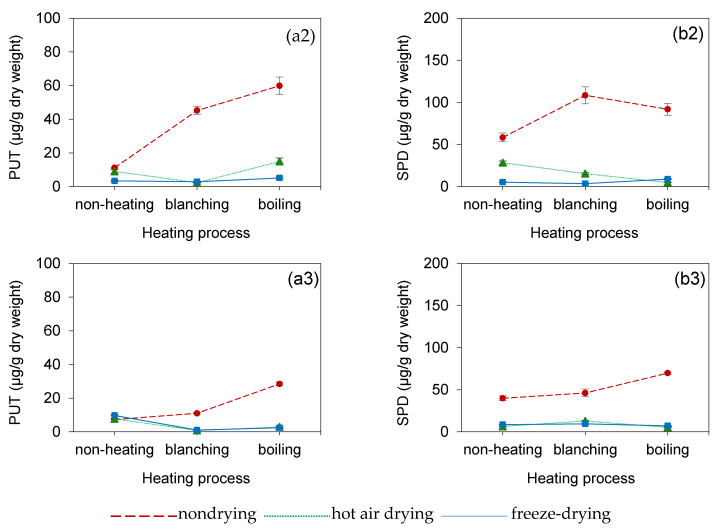
The changes of the PUT content (µg/g of dry weight) in the *H. erinaceus* extracts from different pretreatment processes: (**a1**) whole, (**a2**) ripping, and (**a3**) chopping and the changes of the SPD content (µg/g of dry weight) in the *H. erinaceus* extracts from different pretreatment processes: (**b1**) whole, (**b2**) ripping, and (**b3**) chopping.

A few reports dealing with a different shapes and sizes influenced the biogenic amine contents; for example, the TYR content was increased two-fold in disc-shaped cheeses compared to bar-shaped cheeses [38]. Some authors reported the size of the sample as a factor that affects the formation of biogenic amines. Latorre-Moratalla et al. [39] suggested that a diameter of about 4.5 cm had higher biogenic amine contents than a diameter of 2.5 cm. in Spanish dry fermented sausages. In the same way, Bover-Cid et al. studied the biogenic amine levels in dried fermented sausages, and the biggest diameter was higher than in the thinnest sausages [40]. These reviews are related to the results of this study. In the case of the nondrying method, the trend of biogenic amine levels in the whole sample (a1 and b1) was the highest compared to ripping (a2 and b2) and chopping (a3 and b3), as shown in Figure 3. The high oxygen accumulation inhibited the production of biogenic amines [41]. Therefore, a bigger size of sample might increase the chance of biogenic amine occurrence by the limitation of accessed oxygen.

The present work also significantly exhibited the effect of the heating process on the PUT and SPD contents in the *H. erinaceus* extract (*p* < 0.05). Several studies have confirmed the heating process being related to the contents of the biogenic amines. Preti et al. [42] suggested that PUT and SPD had higher levels after the boiling of green bean (*Phaseolus vulgaris*) in 24% and 41.5% increments, respectively. Some literature has reviewed that the biogenic amine level significantly increased from 8.7 mg per 100 g of the sample to 13.4 mg per 100 g of the sample after the boiling process of eggplants (*Solanum melongena* L.) [43]. The heating process may enhance the availability of biogenic amine extraction from intercellular molecules and altered cell structures [25]. In the present study, blanching and boiling increased the biogenic amine levels for the nondrying *H. erinaceus* samples (Figure 3).

Conversely, the drying method exhibited a strong effect (*p* < 0.000) on the PUT and SPD contents in the *H. erinaceus* extract. Figure 3 showed a higher level of biogenic amines, both PUT and SPD, from the nondrying samples. There is some literature about the drying method on the biogenic amine content, but no data was reported for *H. erinaceus*. Our study showed that nondrying left a significantly large amount of the biogenic amine levels compared to hot air drying and freeze-drying, as shown in Table 4. High levels of the biogenic amines (PUT and SPD) were clearly shown for the nondrying method (treatments 1, 4, 7, 10, 13, 16, 19, 22, and 25).

The deficiency of the present work on the pretreatment processes induced the alteration of the biogenic amines in *H. erinaceus*. A hypothesis could be assumed that the amount of biogenic amine might be produced during the steps of the storage and pretreatment processes. Normally, the samples were kept at room temperature. Decarboxylase could generate biogenic amines by amino acid decarboxylation due to its optimum temperatures (20–37 °C) [36,44]. The trends of the biogenic amines generally increased after cooking, including grilling, steaming, blanching, and boiling. Heating could inactivate an amine oxidase enzyme, which converted the amines to ammonia and aldehyde. The heating process enhanced the extraction for a more efficient release of the biogenic amines from intercellular proteins and altered cell structures. Similarly, the precursor amino acids can also be easily degraded, leading to the formation of biogenic amines [25,36,42,45]. These amines are concerning, because their presence is an important indicator of food acceptance and/or quality. Biogenic amines should be controlled during all of the food chain, including harvesting, raw material, pretreatment, processing, final product, and storage, to be confident in consumer safety.

Nevertheless, different types of drying may affect biogenic amines. There is a possibility that a decrease of the pressure in the process of freeze-drying can lead to enzyme inactivation, including decarboxylase, and the prompting of molecule metabolism [45]. Novella-Rodríguez et al. [45] studied the effects of eliminating the pressure processes on the biogenic amine contents in goat cheese. The results showed that the TYR content significantly dropped from 10.3 to 1.6 mg/L. Besides, the freeze-drying process used a low temperature that also helps to inhibit the microorganism, which caused the origin of the decarboxylase enzyme [46]. Freeze-drying is considered one of the best effective drying methods for food preservation, which can produce high-value dried products with good sensory qualities and high levels of nutrient retention. However, freeze-drying requires a high energy-consuming operation, takes a long-time process cycle, and uses expensive equipment [47,48]. Therefore, hot air drying, a common method of dehydration, is a better alternative. It can retain the bioactive compound and still preserve the quality of the sample, i.e., shape, aroma, taste, and color. Especially, this conventional method is super low-cost compared with freeze-drying [49,50].

β-glucan is mainly represented as a group of bioactive compounds in mushrooms. β-glucan can promote human health with anticarcinogen, antitumor, anti-inflammatory, antiallergenic, antimicrobial, and immunological activity [3,51,52,53,54,55]. Figure 1 shows the quantity of the β-glucan in the *H. erinaceus* extracts in different pretreatment processes. This study found that the β-glucan contents of the extracts from *H. erinaceus* treated by different processes ranged from 173.99–552.52 mg/g dry weight. The amount of β-glucan in this study was similar to a previous survey that found 198 ± 65 mg/g of the sample or 20% dry weight in *H. erinaceus* [51] and detected β-glucan in the range of 10–45% dry weight in shiitake mushroom (*L. edodes*) [52]. The treatments with the drying methods (both hot air drying and freeze-drying) clearly showed high levels of β-glucan contents. The amount of antioxidant activity of the Trolox equivalent also showed a high level in Figure 2.

The contents of the biogenic amines (PUT and SPD), β-glucan, and antioxidant activity were considered to perform low levels of biogenic amines and retention levels of β-glucan and antioxidant activity in *H. erinaceus* extracts from 27 pretreatment processes. Considering both of the biogenic amine (PUT and SPD) contents, treatments 3, 6, 8, 9, 12, 15, 18, 20, 21, 23, 24, 26, and 27 showed no significant differences in their low biogenic amine contents (Table 4). These low biogenic amines of the pretreatment processes were considered together with high levels of β-glucan and antioxidant activity, in which treatments 8, 20, and 23 were suitable pretreatment processes for this work. In this study, treatment 20 (chopping/non-heating/hot air drying) is a better choice for the pretreatment process of the *H. erinaceus* extracts, owing to the fact that chopping, the smaller size of the sample, can decrease the biogenic amines that occur and the non-heating process can also help to diminish the formation of biogenic amines.

## 5. Conclusions

This study generated information on the biogenic amines of the *H. erinaceus* extract in order to understand better the effect of the pretreatment process on biogenic amines for monitoring them and improving some bioactive compounds, such as β-glucan and antioxidant activity. This can guarantee the use of suitable raw materials in order to limit the biogenic amines in an end product and assure a better quality. Our present study concluded that treatment 20 (chopping/non-heating/hot air drying) should be suggested as a choice for the pretreatment process. This pretreatment process not only indicated low levels of biogenic amines of the *H. erinaceus* extract but also presented bioactive compounds such as β-glucan and antioxidant activity and was easy to implement and cost-saving.

## Figures and Tables

**Table 1:** The codes of an independent variable on the factorial experimentations for the biogenic amine contents.

Independent Variables	Code Levels		
	−1	0	1
Sample size (X_1_)	whole	ripping	chopping
Heating process (X_2_)	non-heating	blanching	boiling
Drying method (X_3_)	nondrying	hot air drying	freeze drying

**Table 2:** The gradient program of mobile phase A and mobile phase B for the separation of biogenic amines in the extract from *H. erinaceus*.

Operation	Time (Min)	Mobile Phase (%)	
		A	B
elution	0	70	30
	10	70	30
	25	0	100
return	28	70	30
equilibration	35	70	30

**Table 3:** Summary results related to the post-column derivatization HPLC method validation of the biogenic amines in the *H. erinaceus* extract.

Validation Parameters				PUT	SPD
Linearity	R^2^			0.9995	0.9981
Accuracy	Recovery (%)		80%	105.94	101.62
			100%	106.77	100.47
			120%	107.76	101.75
Precision	RSD (%)	Intraday	80%	5.86	5.36
			100%	5.26	4.66
			120%	4.27	4.32
		Inter-day	80%	4.55	4.2
			100%	5.72	4
			120%	4.47	4.22
LOD (mg/mL)				0.01	0.55
LOQ (mg/mL)				0.04	1.67

**Table 4:** The factorial experimentations of the three factors for the contents of the biogenic amines (µg/g dry weight) in the *H. erinaceus* extract.

Treatment	Independent Variables			Biogenic Amines Content ^1^	
				(µg/g Dry Weight)	
	Sample Size	Heat Process	Drying Method	PUT	SPD
1	−1	−1	−1	10.32 ± 0.56	102.52 ± 5.20
2	−1	−1	0	21.95 ± 1.69	31.10 ± 3.08
3	−1	−1	1	4.60 ± 0.18 *	8.95 ± 0.71 *
4	−1	0	−1	83.16 ± 7.82	137.97 ± 4.93
5	−1	0	0	14.13 ± 1.22	6.73 ± 0.62 *
6	−1	0	1	2.84 ± 0.49 *	13.23 ± 1.00 *
7	−1	1	−1	70.43 ± 5.86	148.74 ± 5.06
8	−1	1	0	7.96 ± 0.66 *	9.37 ± 1.47 *
9	−1	1	1	2.52 ± 0.38 *	10.87 ± 0.21 *
10	0	−1	−1	11.24 ± 0.32	58.40 ± 5.20
11	0	−1	0	9.00 ± 1.04	28.39 ± 2.14
12	0	−1	1	3.44 ± 0.37 *	5.29 ± 0.56 *
13	0	0	−1	45.22 ± 2.49	108.43 ± 10.05
14	0	0	0	2.36 ± 0.16 *	15.48 ± 0.87
15	0	0	1	2.93 ± 0.28 *	3.53 ± 0.35 *
16	0	1	−1	59.90 ± 5.19	91.90 ± 7.02
17	0	1	0	14.98 ± 2.00	4.90 ± 0.15 *
18	0	1	1	5.12 ± 0.62 *	8.92 ± 0.75 *
19	1	−1	−1	7.27 ± 0.08 *	39.89 ± 2.78
20	1	−1	0	7.69 ± 0.44 *	6.49 ± 0.18 *
21	1	−1	1	9.73 ± 0.23	8.56 ± 0.31 *
22	1	0	−1	11.02 ± 0.44	46.14 ± 4.63
23	1	0	0	0.81 ± 0.11 *	12.95 ± 0.44 *
24	1	0	1	1.04 ± 0.12 *	9.45 ± 0.58 *
25	1	1	−1	28.45 ± 1.13	69.80 ± 1.30
26	1	1	0	2.92 ± 0.56 *	5.34 ± 0.44 *
27	1	1	1	2.41 ± 0.24 *	7.14 ± 0.38 *

^1^ The values were expressed as the mean ± standard deviation in triplicate. * Represent the same group with a low content according to Tukey’s HSD test *(p* < 0.05) among the same column.
